# DARPins bind their cytosolic targets after having been translocated through the protective antigen pore of anthrax toxin

**DOI:** 10.1038/s41598-023-34647-1

**Published:** 2023-05-17

**Authors:** Lukas Becker, Andreas Plückthun

**Affiliations:** Department of Biochemistry, University of Zurich, Winterthurerstr. 190, 8057 Zurich, Switzerland

**Keywords:** Protein folding, Drug delivery, Protein translocation, Biochemical assays, Recombinant protein therapy

## Abstract

Intracellular protein–protein interactions in aberrant signaling pathways have emerged as a prime target in several diseases, particularly cancer. Since many protein–protein interactions are mediated by rather flat surfaces, they can typically not be interrupted by small molecules as they require cavities for binding. Therefore, protein drugs might be developed to compete with undesired interactions. However, proteins in general are not able to translocate from the extracellular side to the cytosolic target site by themselves, and thus an efficient protein translocation system, ideally combining efficient translocation with receptor specificity, is in high demand. Anthrax toxin, the tripartite holotoxin of *Bacillus anthracis*, is one of the best studied bacterial protein toxins and has proven to be a suitable candidate for cell-specific translocation of cargoes in vitro and in vivo. Our group recently developed a retargeted protective antigen (PA) variant fused to different Designed Ankyrin Repeat Proteins (DARPins) to achieve receptor specificity, and we incorporated a receptor domain to stabilize the prepore and prevent cell lysis. This strategy had been shown to deliver high amounts of cargo DARPins fused behind the N-terminal 254 amino acids of Lethal Factor (LF_N_). Here, we established a cytosolic binding assay, demonstrating the ability of DARPins to refold in the cytosol and bind their target after been translocated by PA.

## Introduction

Intracellular protein–protein interactions in aberrant signaling pathways have emerged as a prime target in several diseases, particularly cancer^1–3^. Direct cell-specific delivery of highly specific inhibitory molecules would provide an efficient and selective way of targeting only aberrant pathways within a cell in a desired tissue. Most therapies today rely on the inhibitory function of cell-permeable small molecules^4,5^. Those, however, cannot be made cell-specific and many protein–protein interaction surfaces are large, rather flat and hydrophobic, lacking a binding pocket for small molecules, and thus leaving many protein–protein interaction surfaces undruggable^6,7^.

Advances in the generation of binding molecules based on alternative binding scaffolds, such as Designed Ankyrin Repeat Proteins (DARPins), have allowed the efficient generation of small binding proteins against virtually any target^8^. DARPins, unlike antibodies, do not require disulfides for stability, and have been demonstrated to fold well when expressed in the cytoplasm of many cells^8–10^. Since proteins, however, are not able to translocate from the extracellular side to the cytosolic target site by themselves, an efficient protein translocation system, ideally combining efficient translocation with receptor specificity, is in high demand. Bacterial protein toxins have naturally evolved to translocate their toxic cargo protein to the cytosol in a cell-specific manner^6,11^. Our group and others have adapted such toxins to deliver non-native cargoes to cells expressing various receptors^11–17^.

**Figure 1:**
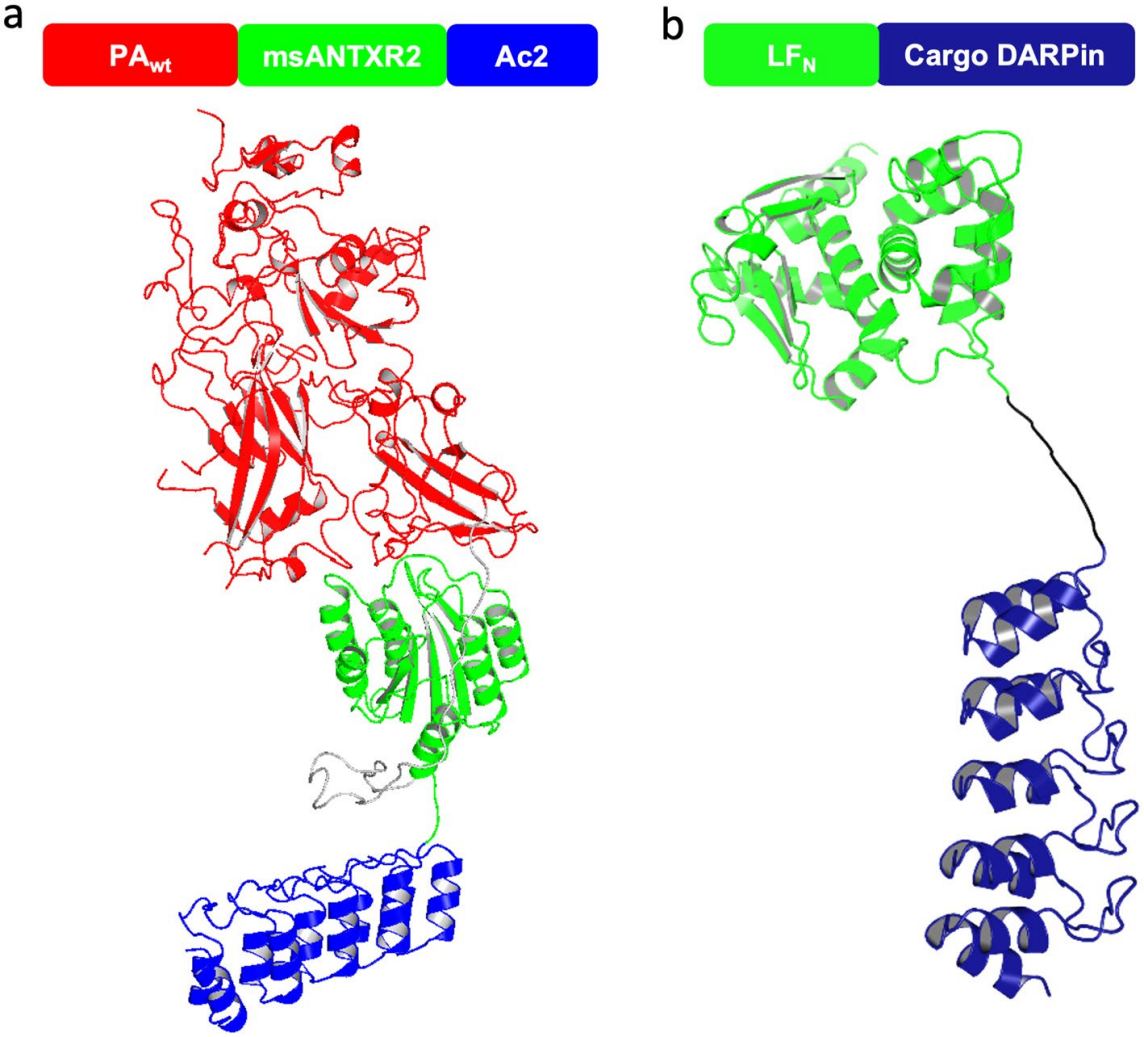
(**a**) Schematic diagram and structural model of pore component, consisting of protective antigen (red), a receptor domain to stabilize it and prevent premature activation (green) and the retargeting DARPin (blue), providing cell specificity. (**b**) Schematic diagram and structural model of translocated payload, consisting of N-terminal 254 residues of lethal factor (green), which interacts with protective antigen, and fused DARPin (blue), which binds to a cytosolic target.

Anthrax toxin, the tripartite holotoxin of *Bacillus anthracis*, is one of the best studied bacterial protein toxins and has proven to be a suitable candidate for cell-specific translocation of cargoes alternative to its own toxic component in vitro and in vivo^12,18–20^. Our group recently developed a retargeted protective antigen (PA) variant which is fused to a retargeting DARPin binding to the receptor of choice, here EpCAM bound by the DARPin Ac2, to achieve receptor specificity, and on incorporating a receptor domain to stabilize the prepore and prevent cell lysis. Using the translocation domain of anthrax toxin, this system was shown to be able to deliver high amounts of cargoes^19,21^. Since DARPins can be easily selected to bind to virtually any target and since they have been shown to be effective within the cytosol, they have been used by us and others as alternative cargo molecules by fusing the cargo DARPin behind the N-terminal 254 amino acids of LF (LF_N_). Since LF_N_ and DARPin are flexibly linked, it is reasonable to assume that the DARPin retains its binding characteristics in this context^8,19,22^. The two components of the transport system are depicted in Fig. 1.

Cargo molecules, translocated via anthrax toxin, have to unfold to penetrate through the narrow PA-channel and once in the cytosol, they must refold to bind to their cytosolic target^23^. Previously, DARPin translocation has been demonstrated via the BirA assay, a western blot-based assay that shows biotinylation of the cargo DARPin on an avi tag by BirA, a biotin ligase from *E. coli,* which has been stably expressed in the cytosol of the mammalian cells under study^24^. As an alternative readout, the toxicity by co-delivering diphtheria toxin (DTA) was reported^22^. Both methods conclusively show the presence of the cargo but not its function, and a direct readout for cytosolic refolding and activity of the DARPin has been missing^19,24^.

Since DARPins must be in the folded state to bind their target^8^, we established here an ELISA-based cytosolic binding assay and thereby prove the ability of DARPins to refold in the cytosol and bind their cytosolic target after having been translocated through the PA channel.

## Results

Previously, we showed that the delivery of various DARPin cargoes to the cytosol of Flp-In 293-EpCAM-BirA cells is dependent on thermodynamic stability, probably due to the required unfolding step when traversing the pore. We carried out these experiments using an assay employing cells stably overexpressing the epithelial cell adhesion molecule (EpCAM) as the receptor to be targeted, and *E.* *coli* biotin ligase (BirA), stably expressed in the cytosol, to biotinylate the avi tag attached to the cargo once it has reached the cytosol^25^. We thus confirmed the presence of cytosolic DARPin cargoes, and thereby the successful translocation, by utilizing the BirA assay previously developed^24^. However, the BirA assay cannot detect whether the translocated DARPin has refolded and is able to bind to its cytosolic target.

**Figure 2:**
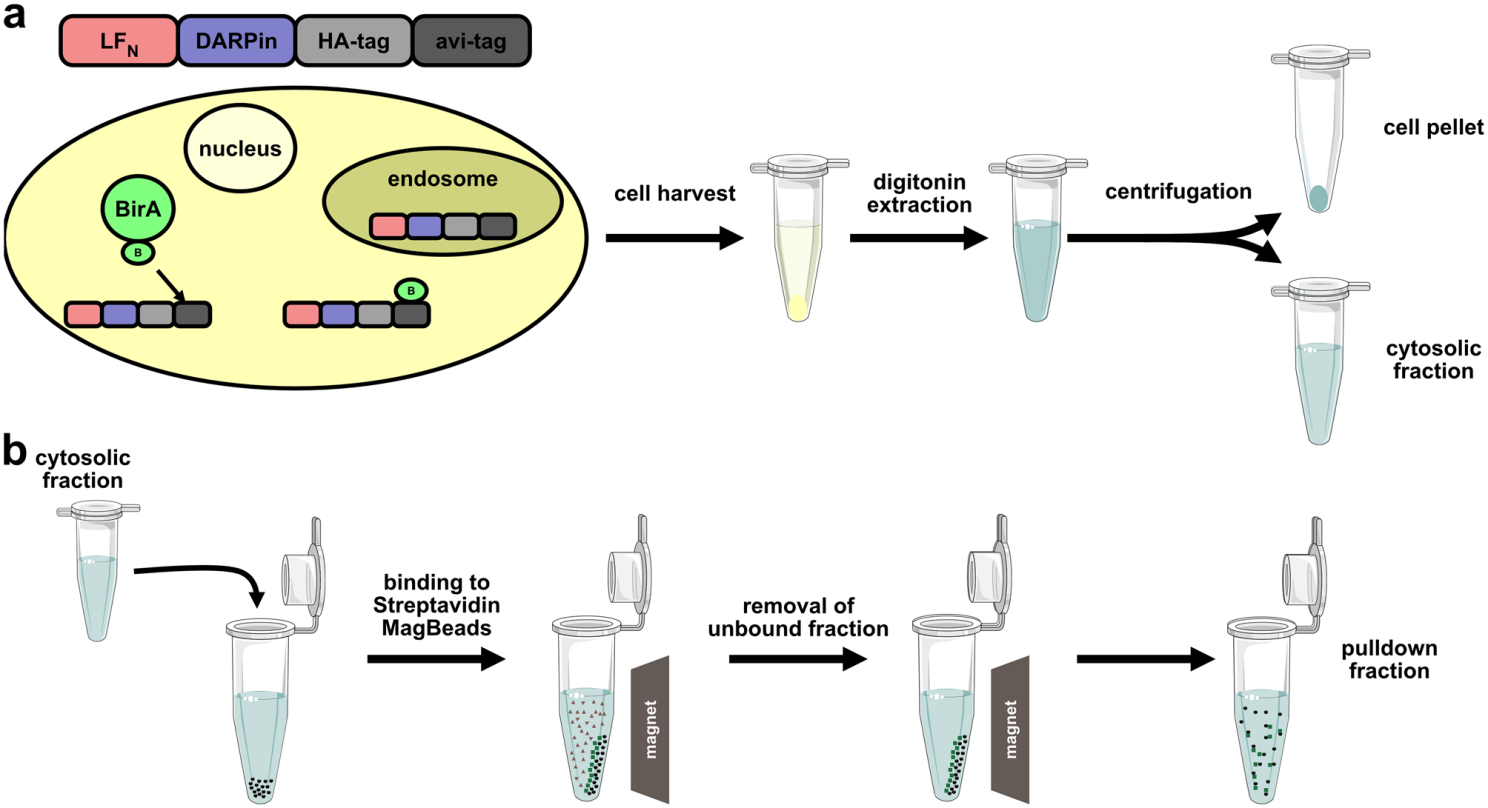
Scheme of anthrax toxin-based delivery with subsequent pulldown to test successful refolding. (**a**) LF_N_-DARPin-HA-tag-avi-tag gets delivered to the cytosol and biotinylated by cytosolically present BirA. Protein stuck in the endosome does not get biotinylated. Cells are harvested, extracted with digitonin, and the cytosolic fraction is separated from the residual pellet by centrifugation. (**b**) The cytosolic fraction is added to streptavidin magnetic beads and the unbound fraction is removed. The pulldown fraction is then analyzed further via western blotting and ELISA.

Here, we set out to perform a pulldown assay detected by ELISA to confirm cytosolic binding of the translocated DARPins to their cytosolic targets. Since DARPins need to be in the folded state for binding^8^, we can use this assay to deduce their correct folding. We therefore established a digitonin-based extraction protocol of the cytosolic fraction of targeted cells with a subsequent pulldown of the translocated LF_N_-DARPin cargo^22^. The cargo additionally carries both an HA-tag and an avi-tag fused to its C-terminus (Fig. 2). Therefore, cells were incubated with respective delivery components (protective antigen targeting EpCAM and cargo DARPin fused to LF_N_ and the detection tags). Cells are then harvested, and the cytosolic fraction extracted with digitonin extraction buffer (Fig. 2a). To pull down the translocated LF_N_-DARPin cargo, biotinylated via the resident BirA, streptavidin-carrying magnetic beads were incubated with the cytosolic fraction. After removal of the unbound fraction, the pulldown fraction was used for further analysis (Fig. 2b).

**Figure 3:**
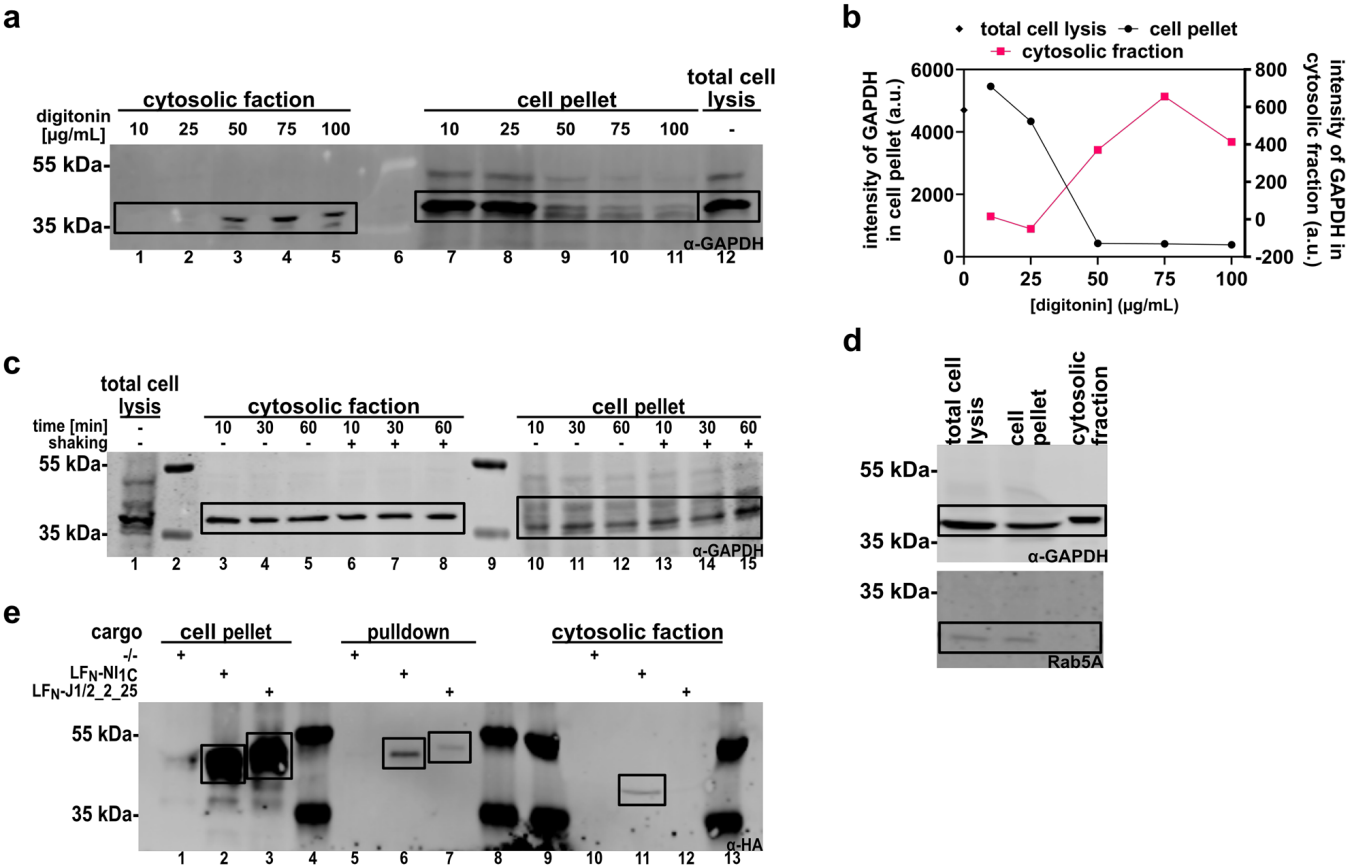
Western blot analysis of Flp-In 293-EpCAM-BirA for cytosolic extraction with digitonin. (**a**) Western blot of GAPDH from Flp-In 293-EpCAM-BirA cells, incubated with 10–100 µg/mL digitonin for 10 min, 4 °C. Cytosolic fractions (lane 1–5) were separated from other cellular compartments (lane 7–11) by centrifugation. Cells labelled “total cell lysis” (lane 12) were incubated directly in Laemmli sample buffer. All samples were analyzed via western blot, stained with anti-GAPDH. (**b**) Quantification of western blot bands from (**a**). (**c**) Western blot of GAPDH from Flp-In 293-EpCAM-BirA cells incubated with 50 µg/mL for 10–60 min on ice without shaking (lane 3–5, 10–12) or with shaking at 4 °C (lane 6–8, 13–15). Cytosolic extracts (lane 3–8) and other cellular compartments (lane 10–15) were analyzed and stained with anti-GAPDH. Cells labelled “total cell lysis” were directly incubated in 1 × Laemmli sample buffer. Lanes 2 and 9 were loaded with protein MW marker. (**d**) Western blot of GAPDH and Rab5A from Flp-In 293-EpCAM-BirA cells incubated in 50 µg/mL digitonin extraction buffer for 10 min and analyzed via western blot, staining for GAPDH (cytosolic fraction) and anti-Rab5A (endosomal fraction). (**e**) Western blot analysis of HA-tagged DARPins in Flp-In 293-EpCAM-BirA incubated with 50 nm PA_wt_-sANTXR-Ac2 and 500 nM LF_N_-NI_1_C (lanes 2, 6, 11) or LF_N_-J1/2_2_25 (lanes 3, 7, 12). DARPin cargoes were pulled down from the cytosolic fraction via streptavidin magnetic beads (lanes 5–7) and the beads were treated with Laemmli buffer. The remaining cytosolic fraction after pulldown (lanes 10–12), as well as the residual cell pellet of the digitonin extraction (lanes 1–3) are shown in addition. Lanes 4, 8–9 and 13 were loaded with protein ladder. The western blot was stained with anti-HA-tag for LF_N_-DARPin detection. High concentrations of digitonin in samples 10–12 lead to a different running behavior of analyzed samples, resulting in protein bands appearing at lower molecular weight.

First, we needed to determine a suitable digitonin concentration for cytosolic extraction, which is efficient but leaves endosomal compartments fully intact, as their leakage would distort the determination of location and thus the binding assay. The western blot (Fig. 3a) and the quantification of it (Fig. 3b) show cytosolic fractions of Flp-In 293-EpCAM-BirA cells incubated with increasing concentrations of digitonin. We found that a concentration of 50 µg/mL digitonin or higher is needed for efficient cytosolic extraction. Furthermore, we tested the digitonin incubation time with 50 µg/mL and found no differences between 10–60 min of incubation, enabling us to perform the assay with 10 min incubation on ice (Fig. 3c). Since higher concentrations than 50 µg/mL of digitonin did not tremendously increase the cytosolic extraction, we stained a western blot of the cytosolic fraction extracted with 50 µg/mL digitonin for Rab5A, an established endosomal marker^26^, and confirmed that 50 µg/mL of digitonin did not permeabilize the endosomal membrane (Fig. 3d).

We then performed a delivery assay, based on the previously established protocol of the BirA assay^24^; however, instead of generating whole cell lysates, we extracted the cytosolic fraction with the digitonin extraction buffer as described above and separated all other cellular compartments, including endosomes, by centrifugation. The cytosolic fraction was then incubated with streptavidin magnetic beads for 2 h for LF_N_-DARPin cargo pulldown (Fig. 2). To confirm the presence of successfully delivered LF_N_-DARPin cargoes in the pulled-down fraction constituting the digitonin extract of the cytosol, we tested two DARPins, NI_1_C and J1/2_2_25, which previously showed highly efficient cytosolic delivery^25^. DARPin NI_1_C is a consensus designed DARPin with a single internal repeat without target binding^27^, J1/2_2_25 is a target selected NI_2_C DARPin binding to JNK1^9^.

The pulldown of the biotinylated DARPins by streptavidin magnetic beads from the cytosol of targeted cells confirmed the presence of both DARPins in western blots by detecting their HA tag (Fig. 3e), with the DARPin NI_1_C being delivered in much greater quantities than J1/2_2_25. This higher delivery of NI_1_C, found here with the digitonin-based solubilization of the cytosolic fraction, is consistent with the previously found results based on total lysis and detection via avi-tag biotinylation (BirA assay)^25^. It is also consistent with an ELISA-based detection of the HA-tag of pulled down LF_N_-DARPins (Supplementary Fig. S1). Therefore, all three quantification methods confirmed the highly efficient delivery of LF_N_-NI_1_C, being 2.8–4.5 × more efficiently delivered to the cytosol than LF_N_-J1/2_2_25 (Supplementary Fig. S1c). Conversely, this agreement shows that all assays are sufficiently reliable for quantification of translocation.

**Figure 4:**
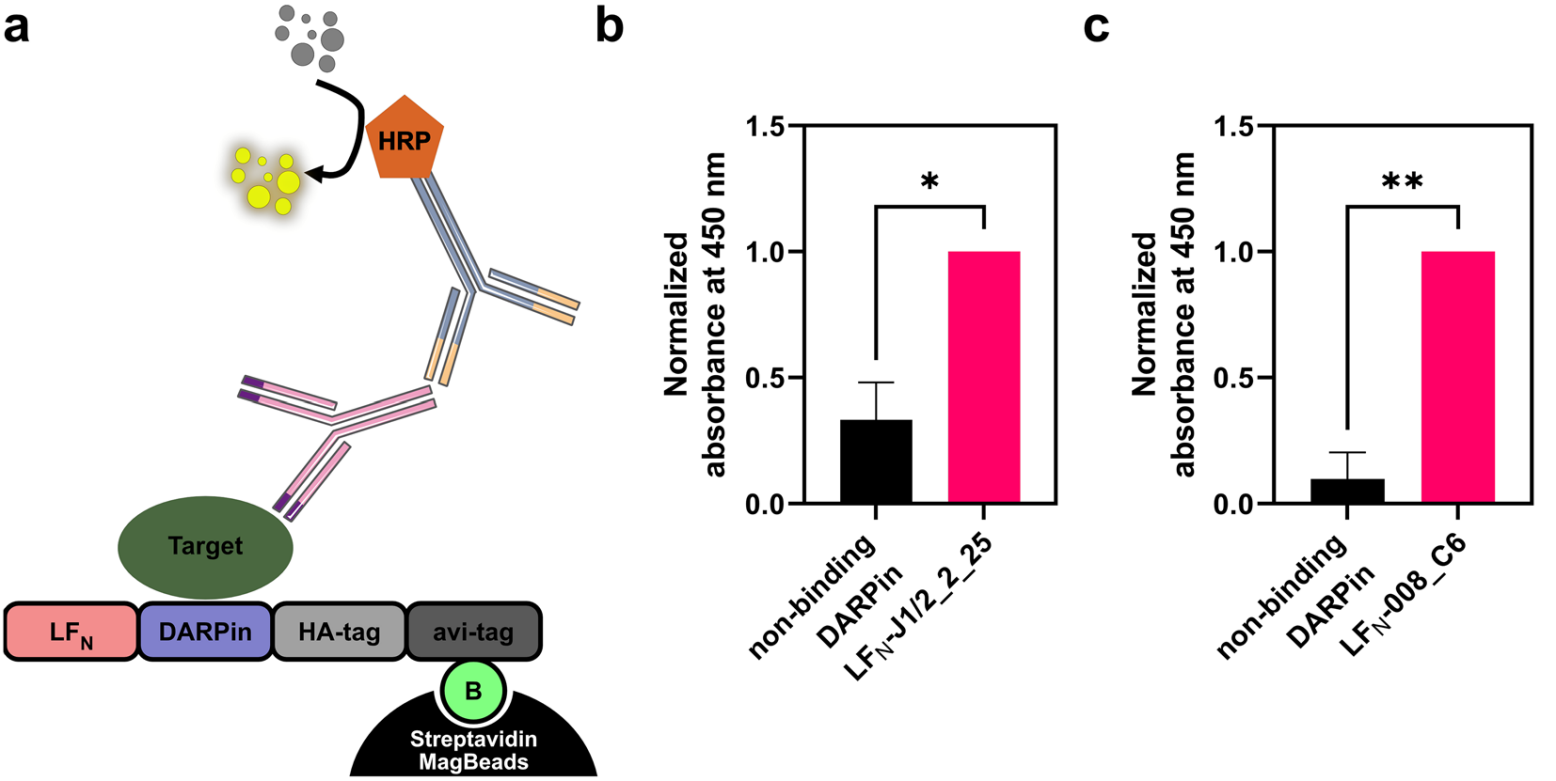
Pulldown ELISA of LF_N_-J1/2_2_25 and LF_N_-008_C6. (**a**) Assay scheme of pulldown ELISA. LF_N_-DARPin is pulled down with streptavidin magnetic beads via the avi-tag biotinylated by cytoplasmic BirA. An anti-JNK1 (**b**) or anti-BCL2 (**c**) primary antibody and an HRP-labelled secondary antibody are used for quantification of pulled down target. (**b, c**) Delivered LF_N_-J1/2_2_25 (**b**) and LF_N_-008_C6 (**c**) show a higher absorbance compared to a non-binding DARPin control when stained with anti-JNK1 (**b**) or anti-BCL2 (**c**) antibody. Values were normalized between 0 (cells only) and 1 (binding DARPin). Statistical analysis: two-tailed unpaired *t* test; *p < 0.05; **p < 0.01. Error bars reflect SEM (n = 3).

In order to detect interaction of DARPin J1/2_2_25, we first stained the western blot in Fig. 3 for its target JNK1, but we could not detect JNK1 pulled down with J1/2_2_25 on this blot (Supplementary Fig. S2). We propose that the signal for JNK1 is below the detection limit on a western blot, taking the weak signal of the western blot band of the LF_N_-J1/2_2_25 construct itself into account and the respective quantification of LF_N_-DARPin cargo translocated to the cytosol (Supplementary Fig. S3). We therefore set out to analyze the pulled down fraction via a more sensitive ELISA-based assay, similar to a recently published delivery assay^28^. Following the pulldown, we incubated the beads, containing the biotinylated DARPin bound to streptavidin, with primary antibody against the DARPin’s target and detected this antibody with an HRP-labelled secondary antibody (Fig. 4a). We measured the absorbance at 450 nm of the LF_N_-JNK1 binding construct (LF_N_-J1/2_2_25) and the non-binding LF_N_-NI_1_C as a control. A cells-only control was included to determine the background absorbance for normalization, while the LF_N_ fused to the binding DARPin, i.e. J1/2_2_25 was set as the maximum absorbance.

The JNK1-targeted DARPin showed increased absorbance in ELISA, compared to the non-binding control, confirming the pulldown of the target, JNK1, and thus it can be reasonably concluded that the DARPin successfully refolded and thus binds its target in the cytosol (Fig. 4b). We then tested a second DARPin-target combination to verify these results. We chose the BCL2 binder 008_C6, which has previously shown cytosolic activity and efficient cytosolic translocation^25,29^. Similar to the LF_N_-J1/2_2_25, the LF_N_-008_C6 construct showed an increase in absorbance compared to the non-binding control (Fig. 4c), consistent with its successful cytosolic refolding and binding to its cognate target BCL2.

## Discussion

In this study, we show for the first time the successful DARPin cargo refolding after translocation to the cytosol with PA via a cytosolic binding assay that is dependent on the folding of the DARPins. We delivered two different target-selected DARPins to the cytosol and tested their capability of binding their cytosolic target in an indirect pulldown assay, which requires the interaction of the folded DARPin and the folded target, as demonstrated from the crystal structures of the complexes^30,31^. With this assay, we indirectly confirmed successful refolding based on the reasonable assumption that the pulldown only occurs for cytosolically active DARPins, as only folded DARPins bind their targets, and DARPins fold cooperatively^8^.

We first developed a cytosolic extraction protocol based on digitonin, which leaves the endosomal membranes intact and does not solubilize endosomal markers, and found similar extraction conditions as previously published in other delivery assays^22^. We then proved in an ELISA-based assay that DARPins are capable of binding their target in the cytosol after being translocated by anthrax toxin, confirming the concept of DARPin-based cytosolic targeting. The assay developed here is very sensitive, but the particular format used requires the use of BirA-expressing cells. While the current format of the assay does not allow for a direct measurement of the binding-active fraction in the cytosol, we assume, due to the beneficial biophysical characteristics of the DARPins^8^, that the level of active DARPins in the cytosol is high.

Previously, our group had shown that DARPins are active in cells in which they have been cytosolically expressed, can bind and inhibit their targets^9,10^. However, DARPin activity after cytosolic translocation via anthrax toxin, comprising unfolding, traversing a narrow pore and refolding and binding to their targets, had not yet been directly shown.

Several other LF_N_-cargoes have been successfully tested for their cytosolic activity. These cargoes were mostly based on other naturally occurring bacterial protein toxins, e.g., *Pseudomonas* exotoxin A or diphtheria toxin A chain and they were tested for their cellular toxicity^13,32,33^. However, the toxic function is a limitation for a generic assay and toxicity might also arise from other sources. Other alternative protein cargoes also showed cytosolic activity after translocation by anthrax toxin^13,34–38^. Nonetheless, direct interaction is the most generically useful assay for a wide variety of targets. In line with these results, it has been shown here that DARPins belong to the proteins that can be delivered to the cytosol via anthrax toxin and regain their cytosolic activity after translocation and refolding the cytosol.

## Conclusion

Here, we confirmed the cytosolic activity of two DARPins after cytosolic translocation with anthrax toxin. With the highly beneficial biochemical characteristics of DARPins we now generated an anthrax toxin-based targeting platform comprising a retargeted PA and LF_N_-cargo fusions, allowing us to target any cytosolic protein with two layers of specificity, one for the cell surface receptor, the other for the cytosolic target.

## Methods

### Cell lines

Flp-In 293 cells, stably overexpressing EpCAM and BirA (Flp-In 293-EpCAM-BirA)^19^ were cultured using DMEM. The medium was supplemented with 10% fetal calf serum and 100 IU/mL penicillin and 100 µg/mL streptomycin.

### Protein expression and purification

The production of His_6_-MBP-PA_wt_-sANTXR-Ac2 and His_6_-MBP-LF_N_-DARPin-HA-tag-avi-tag cargo constructs has been described before^19,21^. Briefly, protective antigen and lethal factor fusion proteins were expressed in soluble form in the cytoplasm of *E. coli* BL21. Purification was achieved via immobilized metal ion affinity chromatography (IMAC) for all constructs. Fusions between MBP and LF-DARPin constructs were cleaved with TEV protease and further purified via reverse IMAC and size-exclusion chromatography. Fusion proteins containing protective antigen were purified directly via size-exclusion chromatography after IMAC^19,21^.

### Delivery of LF_N_-DARPins

For cytosolic delivery of LF_N_-DARPin constructs, the first steps of the BirA assay, previously developed in our lab, were performed up to the cell harvest after delivery^24^. Briefly, 3 × 10^5^ Flp-In 293-EpCAM-BirA cells were seeded in 24-well plates 24 h prior to a 4 h incubation of cells with delivery components. Delivery components, PA_wt_-sANTXR-Ac2 (50 nM) and LF_N_-DARPin (500 nM), were premixed in DMEM medium containing 10% fetal calf serum, 100 IU/mL penicillin and 100 µg/mL streptomycin, 100 µM biotin, and 50 µM MG132. The medium in which cells were seeded in was replaced by the medium containing the delivery components.

### Cytosolic extraction by digitonin

After 4 h incubation of cells with PA_wt_-sANTXR-Ac2 and LF_N_-DARPin, cells were washed with PBS and detached with trypsin–EDTA. For cytosolic extraction, cell pellets were washed with PBS and suspended in 50 µL digitonin extraction buffer (PBS, supplemented with 1% BSA, 50 µg/mL digitonin, 20 µM avi-tag peptide, 0.1 mg/mL DNase, 0.4 mM 4-(2-aminoethyl)benzolsulfonyl fluoride (AEBSF), 10 mM Leupeptin, 1 mM Pepstatin-A), incubated for 10 min on ice, and then centrifuged for 5 min at 16,000*g* at 4 °C. Supernatants were collected for further processing, i.e. western blotting, streptavidin pulldown and target detection ELISA.

### LF_N_-DARPin pulldown via streptavidin magnetic beads

Streptavidin MagBeads (GenScript) were washed 3 × with PBS containing 1% BSA (PBS-B). 10 µL of beads was added to each cytosolic fraction and rotated for 2 h at 4 °C. Then, beads were washed 3 × with PBS-B and either prepared for western blotting or further used for pulldown ELISA.

### Western blot

Cytosolic fractions of the digitonin extraction were analyzed by adding 4 × Laemmli sample buffer (Bio-Rad). Pelleted fractions of digitonin extraction were resuspended in 20 µL 1 × Laemmli sample buffer. Streptavidin MagBeads were resuspended after pulldown in 20 µL of 1 × Laemmli sample buffer, the supernatant of the bead incubation was diluted with 4 × Laemmli sample buffer. Translocated LF_N_-DARPin amounts were quantified via the BirA assay, by titrating known concentrations of fully biotinylated avi-tagged MBP (cytosolic uptake) or LF_N_-J1/2_25-HA-tag-avi-tag (total cellular uptake) on the same blot.

All samples were heated for 10 min at 96 °C. Samples were separated by SDS-PAGE (4–20% Mini-PROTEAN TGX Stain Free Gels, Bio-Rad) and further transferred to PVDF-FL membranes. Membranes were blocked (4 °C, ON) with 1 × Casein Blocking Buffer (Sigma), incubated for 1 h at RT in PBS containing 0.01% Tween 20 (PBS-T) with primary antibodies, rabbit anti-HA (1:1000; Sigma), mouse anti-JNK1 (F-3) (1:1000; SantaCruz), mouse anti-GAPDH (1:1000; SantaCruz) or rabbit anti-Rab5 (1:1000, Cell Signaling). Antibody staining was followed by 3 × 5 min washing with PBS-T and secondary antibody staining with goat anti-rabbit IgG AF680 (1:5000; Invitrogen) or sheep anti-mouse IgG DyLight 800 (1:5000; Rockland) with a final 3 × 5 min washing step before imaging. Western blots were imaged with a LI-COR Odyssey CLx instrument and band intensities were quantified using the Image Studio Lite (LI-COR). Uncropped blots are shown in Supplementary Fig. S4 and summarized in Supplementary Table ST1.

### Pulldown ELISA

After pulldown, beads were incubated with rabbit anti-HA (Sigma), mouse anti-BCL2 (Cell Signaling) or mouse anti-JNK1 (F3) (SantaCruz) antibodies 1:200 overnight at 4 °C while rotating. Then, beads were washed 3 × with PBS-B and incubated with horseradish peroxidase coupled goat anti-mouse antibodies (Pierce) or goat anti-rabbit antibodies (Cell Signaling Technology) 1:5000 for 2 h at 4 °C while rotating. Beads were then washed 3 × with PBS-B and incubated with 75 µL 1-Step Ultra TMB-ELISA Substrate Solution (Thermo Scientific) for 15–30 min at RT while shaking. 75 µL of 2 M sulfuric acid was added and the absorbance at 450 nm was measured on a Tecan plate reader.

## Supplementary Information

Supplementary Information.

## Data Availability

All data is included in this manuscript and its supplementary files.

## Abbreviations

PA: Protective antigen
DARPin(s): Designed ankyrin repeat protein(s)
LF_N_: Lethal factor 1–254
EpCAM: Epithelial cell adhesion molecule
BirA: Biotin ligase derived from *E. coli*
AEBSF: 4-(2-Aminoethyl) benzenesulfonyl fluoride
