# Supplementary material for: DARPins bind their cytosolic targets after having been translocated through the protective antigen pore of anthrax toxin
Source: Sci Rep. 2023 May 17;13:8048. doi: 10.1038/s41598-023-34647-1 (PMC10192448; doi:10.1038/s41598-023-34647-1)
Supplement: Supplementary file 1 — Supplementary Information. [file 41598_2023_34647_MOESM1_ESM.pdf]

Supplementary information

DARPins bind their cytosolic targets after having been translocated through the protective antigen pore of anthrax toxin

Lukas Becker[1], and Andreas Plückthun[1*]

[1]Dept. of Biochemistry, University of Zurich, Winterthurerstr. 190, 8057 Zurich, Switzerland

*Address correspondence to plueckthun@bioc.uzh.ch

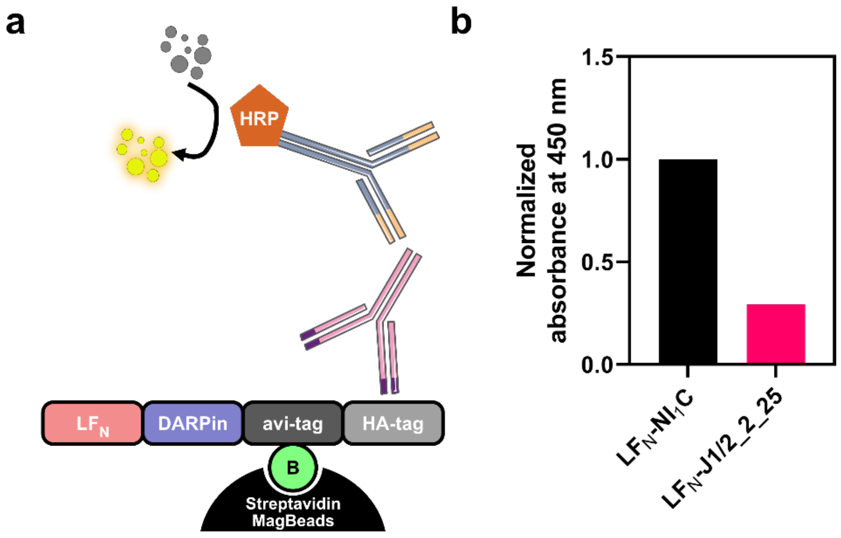

c

Assay	fold increase
ELISA	3.4
Pulldown*	2.8
BirA**	4.5

Figure S1: Pulldown ELISA of LF_N-NI_1C and LF_N-J1/2_2_25 to confirm LF_N-DARPin pulldown. (**a**) Assay scheme of pulldown ELISA confirming the DARPin pulldown quantitated via western blot. LF_N-DARPins are pulled down with Streptavidin magnetic beads via the biotinylated avi-tag. An anti-HA-tag primary antibody and an HRP-labelled secondary antibody are used for quantification of delivered LF_N-DARPin. (**b**) Delivered LF_N-NI_1C shows a higher absorbance at 450 nm compared to LF_N-J1/2_2_25 when stained with anti-HA antibody. Values were normalized between 0 (cells only) and 1 (LF_N-NI_1C); (**c**) Quantitative comparison of different assays for the fold increase of LF-NI_1C compared LF_N-J1/2_2_25. Pulldowns were measured by ELISA and Western blot (*taken from Figure 2), while the BirA assay measures normalized intensity of cytosolic uptake and subsequent cytosolic biotinylation, monitored after total cell lysis (**taken from Becker et al., 2021 [1]).

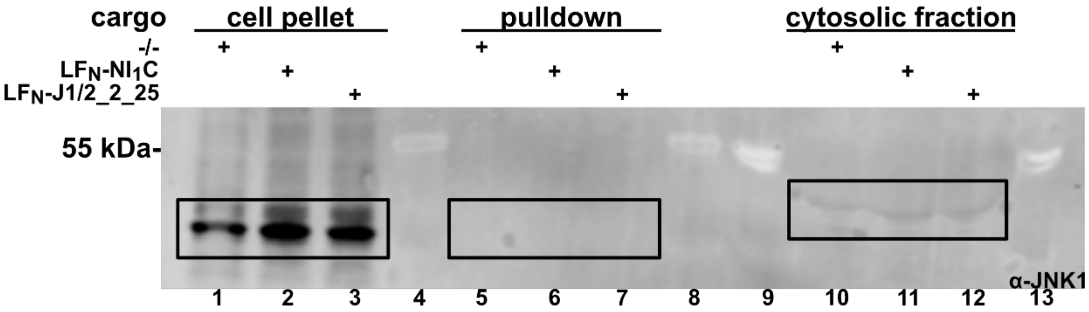

Figure S2: Western blot analysis of Flp-In 293-EpCAM-BirA cells incubated with 50 nm PA_{wt}-sANTXR-Ac2 and 500 nM LF_N-NI_1C or LF_N-J1/2_2_25. LF_N-DARPin cargoes delivered to the cytosol are biotinylated by cytoplasmic BirA and were pulled down from the digitonin extracted cytosolic fraction via Streptavidin magnetic beads. The Western blot was stained with anti-JNK1 antibody. Remaining cytosolic fraction after pulldown, as well as the pellet of the digitonin extraction are shown in addition. JNK1 can be detected in the cell pellet and the remaining cytosolic fraction, but not in the pulldown.

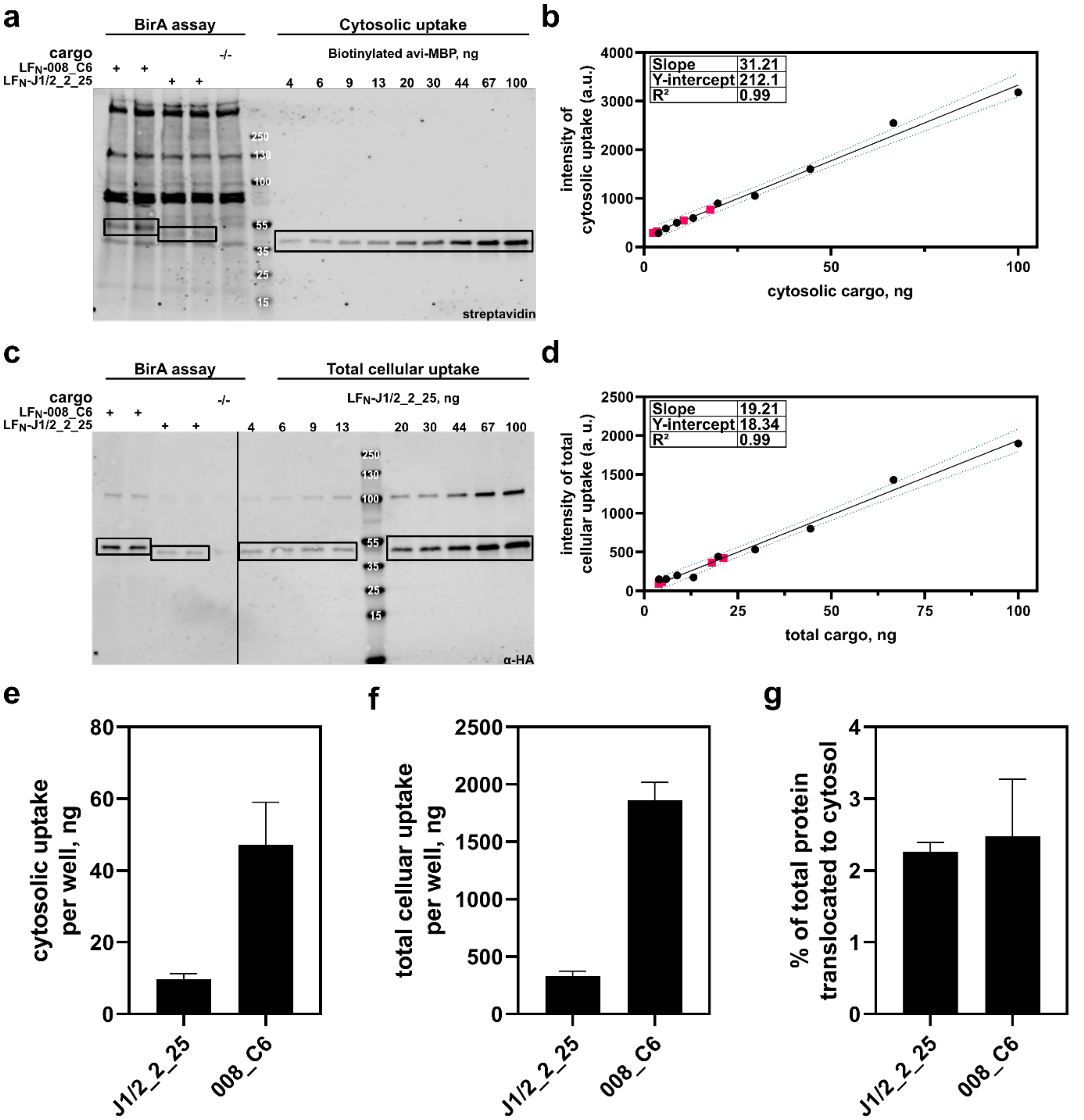

Figure S3: Western blot analysis of cytosolic and total cellular uptake via BirA assay and titration of reference samples. (**a**) BirA assay of Flp-In 293-EpCAM-BirA cells incubated with 50 nm PA_{wt}-sANTXR-Ac2 and 500 nM LF_N-008_C6 and LF_N-J1/2_2_25 to quantify cytosolic uptake. Known concentrations of fully biotinylated avi-tag-MBP was titrated on the same blot for quantification; (**b**) Linear regression of the quantified signals from (a); (**c**) BirA assay of Flp-In 293-EpCAM-BirA cells incubated with 50 nm PA_{wt}-sANTXR-Ac2 and 500 nM LF_N-008_C6 and LF_N-J1/2_2_25 to quantify total cellular uptake. Known concentrations of LF_N-J1/2_2_25 was titrated on the same blot for quantification; (**d**) Linear regression of the quantified signals from (c); (**e**, **f**) Quantified amounts per well of LF_N-DARPin for cytosolic (e) and total cellular uptake (f), error bars represent SEM, n=2; (**g**) Calculated delivery efficiency from (e) and (f).

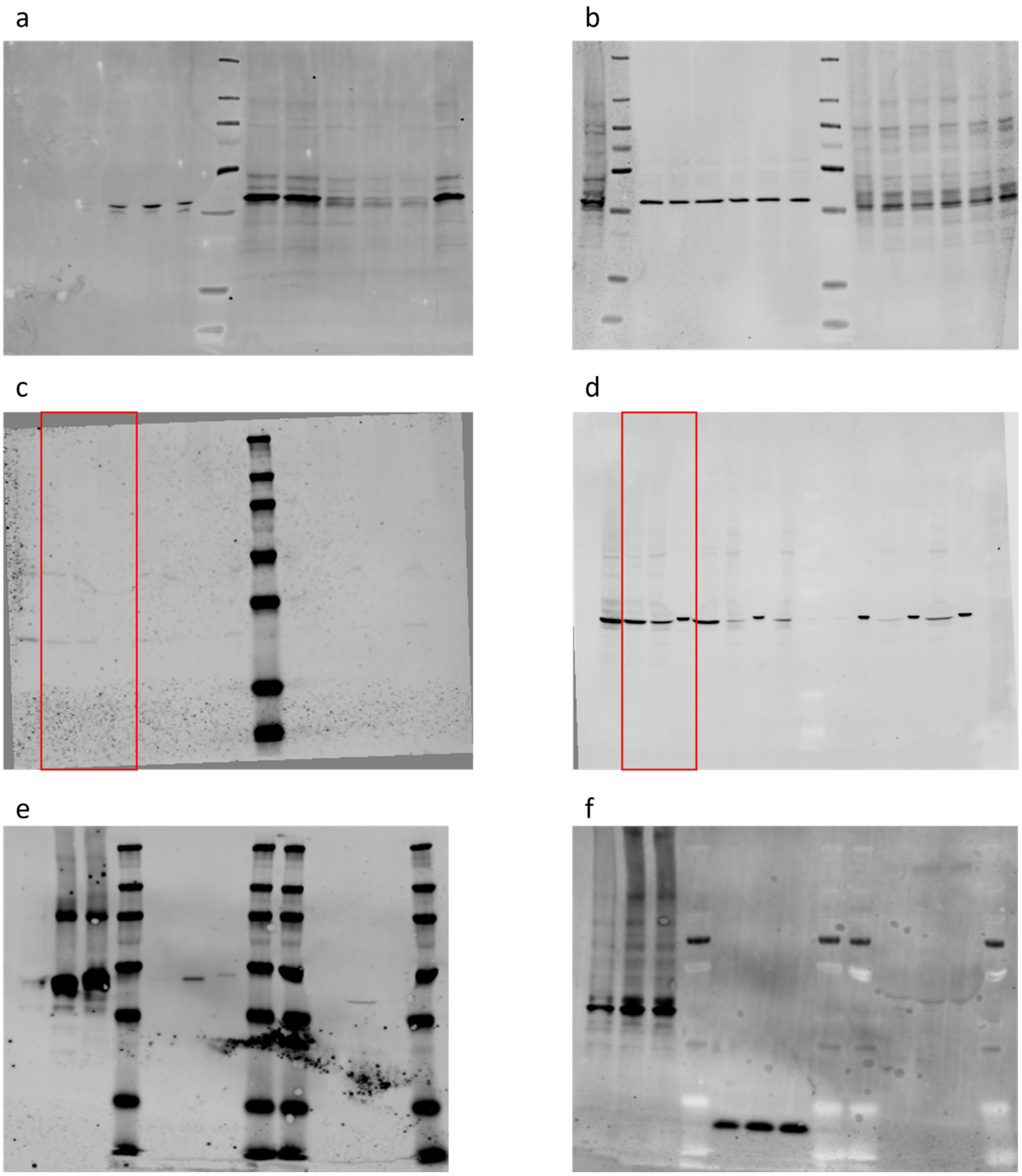

Figure S4: Full Western blot images of uptake assays shown in Figure 2 in the manuscript (a-e) and Supplement Figure S2 (f). Red rectangles (c, d) represent areas of interest for Western blot images cut in the main manuscript (Figure 2d).

Table ST1: Molecular weights of protein constructs analyzed on Western blots.

Protein	MW (kDa)
LF_N-NI_1C	42.7
LF_N-J1/2_2_25	46.2
LF_N-006_C6	51.6
JNK1	48.3

References

1. Becker, L., Singh Badwal, J., Brandl, F., Verdurmen, W. P. R. & Plückthun, A. Thermodynamic stability is a strong predictor for the delivery of DARPins to the cytosol via anthrax toxin. *Pharmaceutics* **13**, 1285 (2021).
